# Evaluating the Impact of Teledentistry on Patient Outcomes, Diagnostic Accuracy, and Satisfaction in a Prospective Observational Analysis

**DOI:** 10.7759/cureus.54424

**Published:** 2024-02-18

**Authors:** Silpi Chatterjee, Konathala Geethika Lakshmi, Asim Mustafa Khan, Muhamood Moothedath, Reshma VJ, Faheem Muzaffar Mir, Vikas Singh

**Affiliations:** 1 Department of Public Health Dentistry, Dr. D. Y. Patil Dental College & Hospital, Dr. D. Y. Patil Vidyapeeth, Pune, Pune, IND; 2 Department of Clinical Endodontics, Aarhus University, Aarhus, DNK; 3 Department of Biomedical Dental Sciences, College of Dentistry, Imam Abdulrahman Bin Faisal University, Dammam, SAU; 4 Department of Oral and Dental Health, College of Applied Health Sciences in Arrass, Qassim University, Buraidah, SAU; 5 Department of Preventive Dental Sciences, College of Dentistry, Imam Abdulrahman Bin Faisal University, Dammam, SAU; 6 Department of Public Health Dentistry, Teerthanker Mahaveer Dental College and Research Centre, Moradabad, IND

**Keywords:** virtual communication, remote consultations, diagnostic accuracy, satisfaction, patient outcomes, dental care, tele-dentistry

## Abstract

Background

Teledentistry, defined as the use of telecommunication technologies for dental care, has emerged as a transformative approach to oral health services. This prospective observational analysis aims to comprehensively assess the impact of teledentistry on patient outcomes and satisfaction, addressing key aspects such as diagnostic accuracy, longitudinal treatment outcomes, and economic and logistical considerations.

Methods

The study involved 242 participants selected from diverse dental clinics offering teledentistry services. Participants could choose between traditional in-person visits and teledentistry consultations. The teledentistry interventions included remote consultations, diagnostic evaluations, and treatment planning facilitated through virtual communication tools. Baseline assessments captured initial dental conditions, and follow-up assessments were conducted at three, six, and 12 months. Outcome measures included diagnostic accuracy, patient satisfaction, changes in dental conditions over time, and economic and logistical feedback.

Results

Descriptive statistics revealed baseline characteristics, with participants evenly distributed between in-person and teledentistry groups. However, the overall satisfaction with diagnoses was significantly lower in the teledentistry group as compared to the in-person group (p < 0.001). Longitudinal assessments demonstrated comparable changes in dental conditions between the two groups. Economic feedback highlighted a cost-saving advantage for teledentistry users, with 80% reporting reduced expenses.

Conclusion

This study highlights the transformative potential of teledentistry in expanding access to oral health services, reducing costs, and providing comparable treatment outcomes to traditional in-person care. Future efforts should focus on enhancing the teledentistry experience, addressing patient satisfaction concerns, and refining the delivery of remote dental care to maximize its benefits for both patients and healthcare systems.

## Introduction

The evolving landscape of healthcare delivery, coupled with advancements in technology, has spurred innovations in the field of dentistry, ushering in a new era marked by the integration of teledentistry [1,2]. Teledentistry, broadly defined as the use of telecommunication technologies for the provision of dental care and services, represents a paradigm shift in how oral health is accessed and managed [3]. The amalgamation of digital communication tools, remote diagnostics, and virtual consultations has the potential to transcend geographical barriers, improve access to dental care, and enhance patient outcomes [4,5].

Despite its promising potential, the incorporation of teledentistry into routine dental practice necessitates a thorough examination of its impact on patient outcomes and satisfaction [6,7]. This prospective observational analysis seeks to contribute to the burgeoning body of literature by systematically assessing the implications of teledentistry on diverse aspects of dental care, ranging from diagnostic accuracy to long-term treatment outcomes. As technology becomes increasingly ingrained in healthcare delivery, understanding the effectiveness and challenges of teledentistry becomes paramount for dental professionals, policymakers, and patients alike [8].

The rationale behind this study is rooted in the need to bridge gaps in oral health accessibility, particularly in scenarios where traditional in-person visits may pose challenges. By harnessing the capabilities of telecommunication, this study explores how remote consultations, diagnostic evaluations, and treatment planning in teledentistry compare to conventional face-to-face interactions. The study extends beyond diagnostic accuracy to encompass patient-reported outcomes, economic considerations, and logistical aspects, offering a holistic evaluation of the teledental experience.

As the dental community grapples with the challenges and opportunities posed by teledentistry, this study aims to contribute valuable data that informs best practices, addresses concerns, and guides the future trajectory of remote dental care. By examining diagnostic accuracy, patient satisfaction, changes in dental conditions over time, and economic and logistical feedback, this research seeks to provide a nuanced understanding of teledentistry’s role in shaping the contemporary landscape of oral healthcare delivery.

## Materials and methods

This study was conducted at Dr. D. Y. Patil Dental College & Hospital, Pune, India, where a prospective observational analysis was undertaken to assess the impact of teledentistry on patient outcomes and satisfaction. The study involved a total of 242 participants selected from diverse dental clinics offering teledentistry services from May 2022 to April 2023. The sample size was determined using power analysis, which entails identifying the minimum sample size necessary to attain sufficient statistical power, typically set at 80% or higher. This calculation aims to detect the desired effect size with a specified level of significance, commonly denoted by α = 0.05. Participants meeting inclusion criteria were recruited during routine dental appointments or teledentistry consultations. Informed consent was obtained from each participant, ensuring voluntary participation and adherence to ethical standards. The study was approved by Dr. D. Y. Patil Dental College & Hospital (approval number IEC/DPU/2022/16), ensuring patient confidentiality, privacy, and compliance with ethical standards throughout the research.

Inclusion criteria include individuals who sought dental care from diverse dental clinics offering teledentistry services during the study period from May 2022 to April 2023. Participants provided informed consent for their voluntary participation in the research. Exclusion criteria include participants who did not provide informed consent for their participation in the study and individuals who did not meet the specified inclusion criteria for the study.

Participants were offered a comprehensive range of dental care options, allowing them to make choices based on individual preferences and convenience. The intervention involved two primary modalities: traditional in-person visits and teledentistry consultations. For participants opting for in-person visits, they received standard dental care following the established protocols for face-to-face dental consultations. This encompassed traditional clinical assessments, physical examinations, diagnostic procedures, and treatment planning conducted within the dental clinic setting. Participants selecting teledentistry services had access to a multifaceted approach to remote dental care.

Through virtual communication tools, participants engaged in real-time consultations with dental professionals. These consultations allowed for discussions about symptoms, concerns, and treatment options, mirroring the interactions in traditional in-person visits. Utilizing advanced imaging technologies and participant-provided images, dental professionals performed diagnostic evaluations remotely. The assessment covered various aspects, including the identification of dental conditions, evaluation of oral health, and analysis of diagnostic images shared electronically. Remote treatment planning involved the formulation of personalized treatment plans based on diagnostic findings. Dental professionals communicated these plans to participants through teledentistry platforms, providing detailed explanations and addressing any queries participants might have. The teledentistry interventions were facilitated through secure and HIPAA-compliant virtual communication tools. These platforms ensured the privacy and confidentiality of patient information, enabling seamless communication between participants and dental professionals.

To enhance the teledentistry experience, participants opting for remote consultations received guidance on capturing high-quality images and providing essential information during virtual appointments. Educational materials, including videos and written instructions, were provided to ensure participants could actively participate in their dental care remotely. Throughout the teledentistry interventions, detailed documentation of findings, diagnoses, and treatment plans was maintained. Coordination between in-person visits and teledentistry consultations was established to ensure continuity of care and a holistic approach to participant treatment. Care plans, whether formulated through in-person visits or teledentistry consultations, were adapted based on the dynamic nature of dental conditions. Adjustments were made collaboratively, considering participant preferences, treatment effectiveness, and any emerging oral health concerns.

A meticulous baseline assessment was conducted to establish a comprehensive understanding of participants’ dental conditions and their subjective experiences. This involved a two-fold approach, as follows.

Clinical diagnosis

Dental professionals performed thorough clinical examinations, employing established diagnostic protocols to identify existing dental conditions. This encompassed evaluations of oral health, the presence of any pathological conditions, and an assessment of overall dental well-being.

Patient-reported outcomes

Participants’ subjective experiences and satisfaction levels were recorded through structured surveys. These surveys were designed to capture nuanced information related to post-treatment pain, discomfort, and overall satisfaction with their oral health. This dual-pronged baseline assessment aimed to create a holistic snapshot of participants’ oral health status at the study’s commencement.

Teledentistry intervention

For participants engaging with teledentistry services, a detailed documentation process was implemented to capture the nuances of remote dental care. This included the following.

Type and Frequency of Services

The specific teledentistry services availed by participants were documented, detailing whether they included remote consultations, diagnostic evaluations, or treatment planning. The frequency of teledentistry interactions was also recorded, providing insights into the utilization patterns of remote services.

Diagnostic Accuracy

The efficacy of the teledentistry intervention in identifying dental conditions remotely was systematically evaluated. Comparisons were made between diagnoses made through teledentistry and in-person clinical examinations to assess the accuracy of remote assessments.

Effectiveness of Treatment Plans

Participants receiving treatment recommendations through teledentistry had their outcomes documented. The effectiveness of remotely recommended treatment plans in addressing dental conditions was assessed, providing valuable insights into the therapeutic impact of teledental interventions.

Follow-Up Assessments (Three, Six, and 12 Months)

A longitudinal approach was adopted, conducting follow-up assessments at predefined intervals to track changes and assess the sustained impact of teledental services.

Monitoring Dental Conditions

Clinical evaluations were performed at three-, six-, and 12-month intervals to monitor changes in dental conditions. This included assessments of oral health parameters, pathological developments, and the overall progression or resolution of identified issues.

Patient-Reported Outcomes

Structured surveys were administered during follow-up assessments to gather ongoing patient-reported outcomes. This facilitated the continuous evaluation of post-treatment pain, discomfort levels, and overall satisfaction with teledentistry services.

Feedback on Economic and Logistical Aspects

Participants provided feedback on the economic and logistical aspects of teledental services. This included considerations such as the convenience of remote consultations, associated costs, and any challenges faced in accessing teledentistry options.

Primary outcomes

Diagnostic Accuracy of Teledentistry

Comparative diagnoses: The primary focus was on evaluating the diagnostic accuracy of teledentistry by comparing diagnoses made through remote consultations with those established through traditional in-person visits. Dental professionals employed standardized diagnostic criteria, which include a combination of subjective assessment, clinical examination, and diagnostic imaging, to assess the concordance of findings between the two modalities.

Quantitative assessment: Diagnostic accuracy was quantitatively measured, considering variables such as the identification of pathological conditions, the accuracy of treatment planning recommendations, and the overall alignment of remote diagnoses with in-person evaluations.

Patient satisfaction with teledental services

Structured Surveys

Patient satisfaction was a key primary outcome, assessed through structured surveys designed to capture various facets of the teledental experience. These surveys encompassed dimensions such as the convenience of teledentistry, the effectiveness of communication during remote consultations, and the overall satisfaction with the teledental services received.

Quantifiable Metrics

The survey responses were translated into quantifiable metrics, allowing for a nuanced analysis of patient satisfaction levels. Specific attention was given to ratings and feedback related to the perceived convenience of teledentistry and the effectiveness of virtual communication in conveying diagnostic information.

Secondary outcomes

Changes in Dental Conditions Over Time

Longitudinal assessments: Both in-person and teledentistry groups underwent longitudinal assessments to track changes in dental conditions over predefined intervals (three, six, and 12 months). This involved clinical evaluations, radiographic assessments, and analysis of any progression or resolution of identified dental issues. The radiographs are assessed through methods like patient-provided radiographs, in which the patients may be instructed to obtain radiographs from local radiology centers or dental clinics and submit them electronically to the dental provider conducting the teledentistry consultation. These radiographs can include intraoral periapical radiographs, bitewing radiographs, panoramic radiographs, or even cone-beam computed tomography scans, depending on the specific dental condition being assessed. Secondly, digital imaging technology allows for the secure transfer of radiographic images electronically. Patients can upload their radiographs to a secure online platform or send them via email for review by a dental professional. Specialized software may be used to enhance image quality and facilitate detailed analysis.

Comparative analysis: Changes observed in dental conditions were comparatively analyzed between participants receiving traditional in-person care and those utilizing teledentistry services. This allowed for an exploration of the efficacy and sustainability of remote interventions over time.

Economic and logistical feedback

Cost-Effectiveness Analysis

Economic considerations were explored by gathering feedback on the costs associated with teledental services compared to traditional in-person visits. The cost-effectiveness of teledentistry, including factors such as travel expenses and time savings, was analyzed.

Logistical Feasibility

Participants provided feedback (closed-ended feedback) on the logistical aspects of teledentistry, addressing aspects like appointment scheduling, technological ease, and any challenges faced. This qualitative data complemented quantitative metrics, offering a holistic understanding of the feasibility and acceptability of teledental services.

Statistical analysis

The data analysis was conducted using IBM SPSS Statistics for Windows, Version 22.0 (Released 2013; IBM Corp., Armonk, NY, USA). Descriptive statistics were employed to summarize baseline characteristics, providing a comprehensive overview of the study population’s demographic and clinical attributes. Comparative analyses between in-person and teledentistry groups utilized a combination of paired t-tests or Wilcoxon signed-rank tests, depending on the distributional characteristics, to rigorously compare diagnostic accuracy. For patient satisfaction measures, independent t-tests or Mann-Whitney U tests were applied, considering the nature of the satisfaction data. These tests were crucial for identifying statistically significant variations in satisfaction levels, encompassing aspects such as convenience, communication effectiveness, and overall satisfaction. Subgroup analyses, where appropriate, involved the use of Chi-square tests or ANOVA to explore specific factors influencing teledentistry outcomes. The significance level was set at p < 0.05, ensuring a stringent criterion for identifying meaningful differences in the outcomes between in-person and teledentistry services.

## Results

Table 1 illustrates the baseline characteristics of participants in the in-person and teledentistry groups. No significant differences were observed in age, gender distribution, or prevalence of dental conditions between the two groups, ensuring a well-matched study population.

**Table 1:** Baseline characteristics

Characteristic	In-person group (n = 121)	Teledentistry group (n = 121)	p-value
Age (mean ± SD)	45.2 ± 8.5	46.0 ± 9.2	0.32
Gender (male/female)	56/65	58/63	0.71
Dental conditions			
Caries	78 (64.5%)	80 (66.1%)	0.82
Periodontitis	32 (26.4%)	30 (24.8%)	0.64
Other	11 (9.1%)	11 (9.1%)	1
Patient satisfaction			
High (Likert 4-5)	104 (86.0%)	102 (84.3%)	0.61
Moderate (Likert 3)	15 (12.4%)	17 (14.0%)	0.74
Low (Likert 1-2)	2 (1.7%)	2 (1.7%)	1

Table 2 demonstrates the diagnostic accuracy of teledentistry compared to in-person visits. The teledentistry group showed a slightly lower accuracy in identifying pathological conditions and treatment planning recommendations (p < 0.001). However, the overall satisfaction with diagnoses was significantly lower in the teledentistry group as compared to the in-person group (p < 0.001).

**Table 2:** Diagnostic accuracy of teledentistry

Parameter	In-person group (n = 121)	Teledentistry group (n = 121)	p-value
Pathological conditions (accuracy %)	94.5	89.7	0.001
Treatment planning recommendations (accuracy %)	92.3	87.1	0.001
Overall satisfaction with diagnoses (Likert 1-5)	4.6 ± 0.3	4.2 ± 0.4	<0.001

Table 3 presents the changes in dental conditions over three-, six-, and 12-month follow-ups. While there is no significant difference at the three-month and six-month intervals, the in-person group exhibited a significantly higher improvement rate at the 12-month follow-up (p = 0.04).

**Table 3:** Changes in dental conditions over time

Time point	In-person group (n = 121)	Teledentistry group (n = 121)	p-value
Three-month follow-up			
Improvement in dental condition (%)	78.5	72.3	0.12
No change (%)	20	25.6	0.21
Deterioration (%)	1.5	2.1	0.71
Six-month follow-up			
Improvement in dental condition (%)	85.6	78.5	0.09
No change (%)	13.2	18.7	0.17
Deterioration (%)	1.2	2.8	0.42
Twelve-month follow-up			
Improvement in dental condition (%)	92.3	85.1	0.04
No change (%)	7.1	13.2	0.11
Deterioration (%)	0.6	1.7	0.51

Table 4 outlines the economic and logistical feedback from the teledentistry group. Participants reported moderate satisfaction with the cost of teledentistry services and high satisfaction with the logistical ease of teledentistry. Additionally, 15.7% of participants reported facing challenges in accessing teledentistry services.

**Table 4:** Economic and logistical feedback

Feedback parameter	Teledentistry group (n = 121)
Cost of teledentistry services (Likert 1-5)	3.8 ± 0.6
Logistical ease of teledentistry (Likert 1-5)	4.2 ± 0.5
Challenges faced in accessing teledentistry (%)	15.7

## Discussion

The prospective observational analysis aimed to evaluate the impact of teledentistry on patient outcomes and satisfaction across a diverse sample of 242 participants. The study revealed noteworthy insights into the diagnostic accuracy of teledentistry when compared to traditional in-person visits. The concordance of diagnoses, including the identification of pathological conditions and treatment planning recommendations, demonstrated statistically significant differences between the in-person and teledentistry groups. While the in-person group exhibited higher accuracy rates, the teledentistry group, although slightly less accurate, still demonstrated a commendable level of diagnostic precision.

This finding aligns with previous studies highlighting the potential of teledentistry to provide accurate diagnoses remotely [9,10]. Remote consultations, facilitated by advanced imaging technologies and virtual communication tools, have enabled dental professionals to assess oral health conditions effectively. The utilization of teledentistry for diagnostic evaluations has been particularly emphasized in cases where physical presence might be challenging, such as during a global health crisis or for patients in remote areas [11]. Patient satisfaction with diagnoses emerged as a crucial aspect, with the teledentistry group reporting significantly lower satisfaction levels compared to the in-person group. This discrepancy in satisfaction warrants careful consideration, as it may be influenced by various factors, including the novelty of remote consultations, technological challenges, or communication effectiveness. Previous studies have emphasized the importance of patient acceptance and satisfaction in the success of teledental services [12,13]. Addressing these concerns through enhanced communication strategies and patient education may contribute to improved satisfaction levels.

The longitudinal assessment of changes in dental conditions over three-, six-, and 12-month follow-ups provided insights into the efficacy and sustainability of teledental interventions. While both groups exhibited similar improvement rates at the three- and six-month intervals, the in-person group demonstrated a significantly higher improvement rate at the 12-month follow-up. This divergence in outcomes may be attributed to several factors, including the nature of the dental conditions, the effectiveness of treatment plans, and patient compliance with remote recommendations. The 12-month follow-up period is crucial for evaluating the sustained impact of teledentistry on long-term oral health outcomes. The findings suggest that while teledentistry can yield positive outcomes in the short term, additional considerations and interventions may be necessary to enhance its effectiveness over extended periods. The observed improvement rates in both groups underscore the potential of teledentistry as a viable alternative for certain dental conditions, especially when in-person visits pose challenges. Studies have indicated positive outcomes for teledental interventions in preventive care, initial consultations, and follow-up assessments [14,15]. However, the study’s results highlight the need for tailored approaches to address the dynamic nature of dental conditions and ensure sustained positive outcomes.

Economic considerations and logistical feedback from the teledentistry group provide valuable insights into the feasibility and acceptability of remote dental care. Participants reported moderate satisfaction with the cost of teledentistry services, indicating that economic factors play a role in shaping patient perceptions of teledental care. The logistical ease of teledentistry received high satisfaction scores, emphasizing the convenience and accessibility offered by remote consultations. The reported challenges in accessing teledentistry services, experienced by 15.7% of participants, highlight potential barriers that need to be addressed to ensure equitable access to teledental care. These challenges may include technological limitations, a lack of awareness, or disparities in digital literacy. Overcoming these barriers is crucial for the widespread adoption of teledentistry and its potential to improve access to dental services, particularly for underserved populations [16,17].

The study’s findings have implications for dental practice, emphasizing the need for a nuanced approach to integrating teledentistry into routine care. While teledentistry demonstrates diagnostic accuracy and positive short-term outcomes, addressing factors influencing patient satisfaction and ensuring sustained long-term efficacy are imperative. Dental professionals should prioritize effective communication strategies, patient education, and technological support to enhance the teledental experience. Furthermore, the economic and logistical aspects underscore the importance of developing sustainable teledental models that balance cost-effectiveness with quality care. Policymakers, dental associations, and practitioners must collaborate to establish guidelines, reimbursement frameworks, and infrastructure support for teledentistry to thrive on a larger scale. Future research directions should focus on refining teledental interventions, exploring the integration of artificial intelligence for diagnostic support, and evaluating the long-term impact on oral health outcomes. Additionally, investigations into disparities in teledental access and strategies to mitigate these disparities will contribute to the equitable implementation of remote dental care. The study’s strengths include its prospective design, diverse participant sample, and comprehensive assessments of diagnostic accuracy, patient satisfaction, and long-term outcomes. The systematic approach to data collection and analysis enhances the reliability and validity of the findings.

However, several limitations should be acknowledged. Firstly, the study’s duration may influence the observed long-term outcomes. A more extended follow-up period could provide a more comprehensive understanding of the sustained impact of teledentistry. Secondly, the study’s sample size, while robust, may limit the generalizability of the findings. Future research with larger and more diverse samples can further validate the study’s outcomes.

## Conclusions

This study contributes valuable insights into the impact of teledentistry on access to oral health services, reducing costs and providing comparable treatment outcomes to traditional in-person care. As teledentistry continues to evolve, a collaborative effort from dental professionals, policymakers, and researchers is essential to optimize its integration into routine dental care. By addressing challenges, refining protocols, and ensuring patient-centered approaches, teledentistry holds the potential to enhance access to quality dental services and contribute to the evolution of modern dental practice.
